# Supplementary material for: Effect of Chain Structure on the Various Properties of the Copolymers of Fluorinated Norbornenes with Cyclooctene
Source: Polymers (Basel). 2023 Apr 30;15(9):2157. doi: 10.3390/polym15092157 (PMC10180767; doi:10.3390/polym15092157)
Supplement: Supplementary file 1 [file polymers-15-02157-s001.zip › polymers-2358735-supplementary.pdf]

Supplementary Materials

Effect of Chain Structure on the Various Properties of the Copolymers of Fluorinated Norbornenes with Cyclooctene

Olga A. Adzhieva [1], Maria L. Gringolts [1,*], Yulia I. Denisova [1], Georgiy A. Shandryuk [1], Ekaterina A. Litmanovich [1,2], Roman Yu. Nikiforov [1], Nikolay A. Belov [1] and Yaroslav V. Kudryavtsev [1,3,4,*]

1 Topchiev Institute of Petrochemical Synthesis, Russian Academy of Sciences, Leninskii pr. 29, 119991 Moscow, Russia; adzhieva@ips.ac.ru (O.A.A.); denisova@ips.ac.ru (Y.I.D.); gosha@ips.ac.ru (G.A.S.); elitmanovich@yandex.ru (E.A.L.); nru@ips.ac.ru (R.Y.N.); belov@ips.ac.ru (N.A.B.)
2 Faculty of Chemistry, Lomonosov Moscow State University, Leninskie Gory 1, Bld. 3, 119991 Moscow, Russia
3 Frumkin Institute of Physical Chemistry and Electrochemistry, Russian Academy of Sciences, Leninskii pr. 31, 119071 Moscow, Russia
4 ESPCI Paris, PSL Research University, 75005 Paris, France
* Correspondence: gringol@ips.ac.ru (M.L.G.); yar@ips.ac.ru (Y.V.K.)

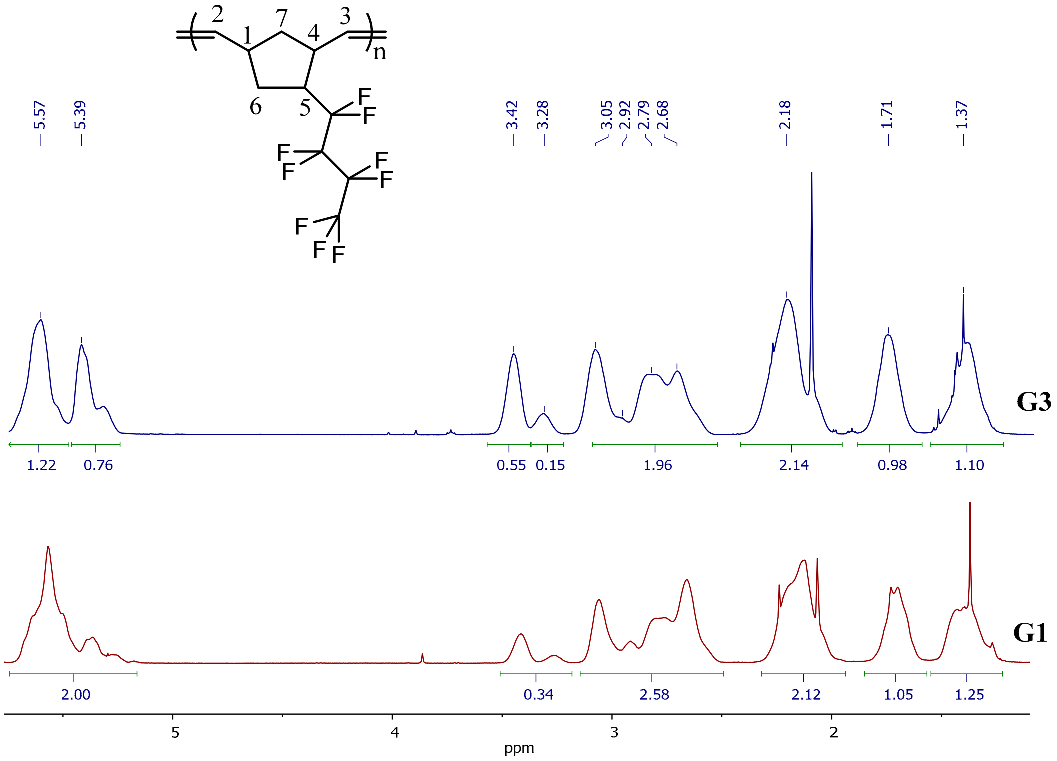

Figure S1. ^{1}H NMR spectra of poly(5-perfluorobutyl-2-norbornene) (PNBF) obtained on the G1 and G3 catalysts at room temperature.

^{1}H NMR (400.1 MHz, $CDCl_3/C_6F_6$) δ, ppm: 5.57 (CH=CH, *trans*), 5.39 (CH=CH, *cis*), 3.42 (HC^5, *cis*, *endo*), 3.28 (HC^5, *cis*, *exo*), 3.05 (HC^5, *trans*, *endo*), 2.92 (HC^5, *trans*, *exo*), 2.79 ($HC^{1,4}$, *cis*), 2.68 ($HC^{1,4}$, *trans*), 2.18 (H_2C^6), 1.71–1.37 (H_2C^7).

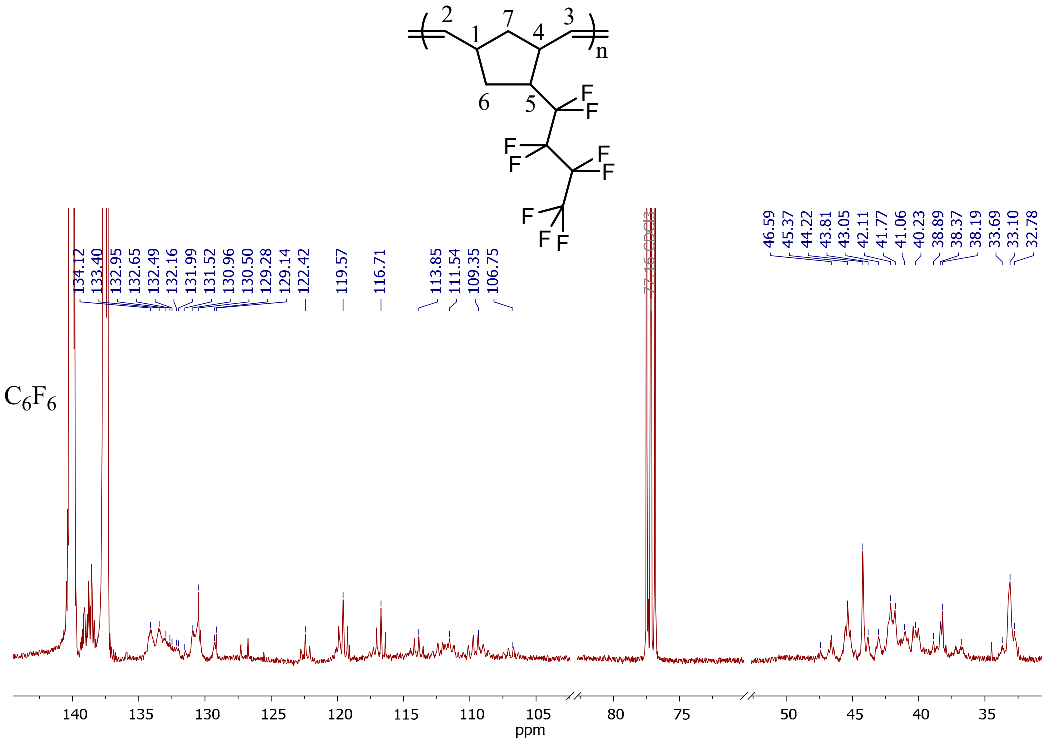

Figure S2. ^{13}C NMR spectrum of poly(5-perfluorobutyl-2-norbornene) (PNBF) at room temperature.

^{13}C NMR (100.6 MHz, $CDCl_3/C_6F_6$) δ, ppm: 134.12–132.16 (C=C, *cis/trans*), 131.99–130.96 (C=C), 130.50 (C^3, *trans*), 129.28, 129.14 (C^3, *cis*), 122.42, 119.57, 116.71, 113.85, 111.54, 109.35, 106.75 (CF), 47.41 (C^5, *exo, cis*) 46.59 (C^5, *exo*, *trans*), 45.37 (C^5, *endo, cis/trans*), 44.22, 43.81 (C^1), 43.05 (C^7), 42.11, 41.77, 41.06, 40.23 (C^4, C^7), 38.89, 38.37, 38.19, 36.78 (C^1, C^4), 33.69–32.78 (C^6).

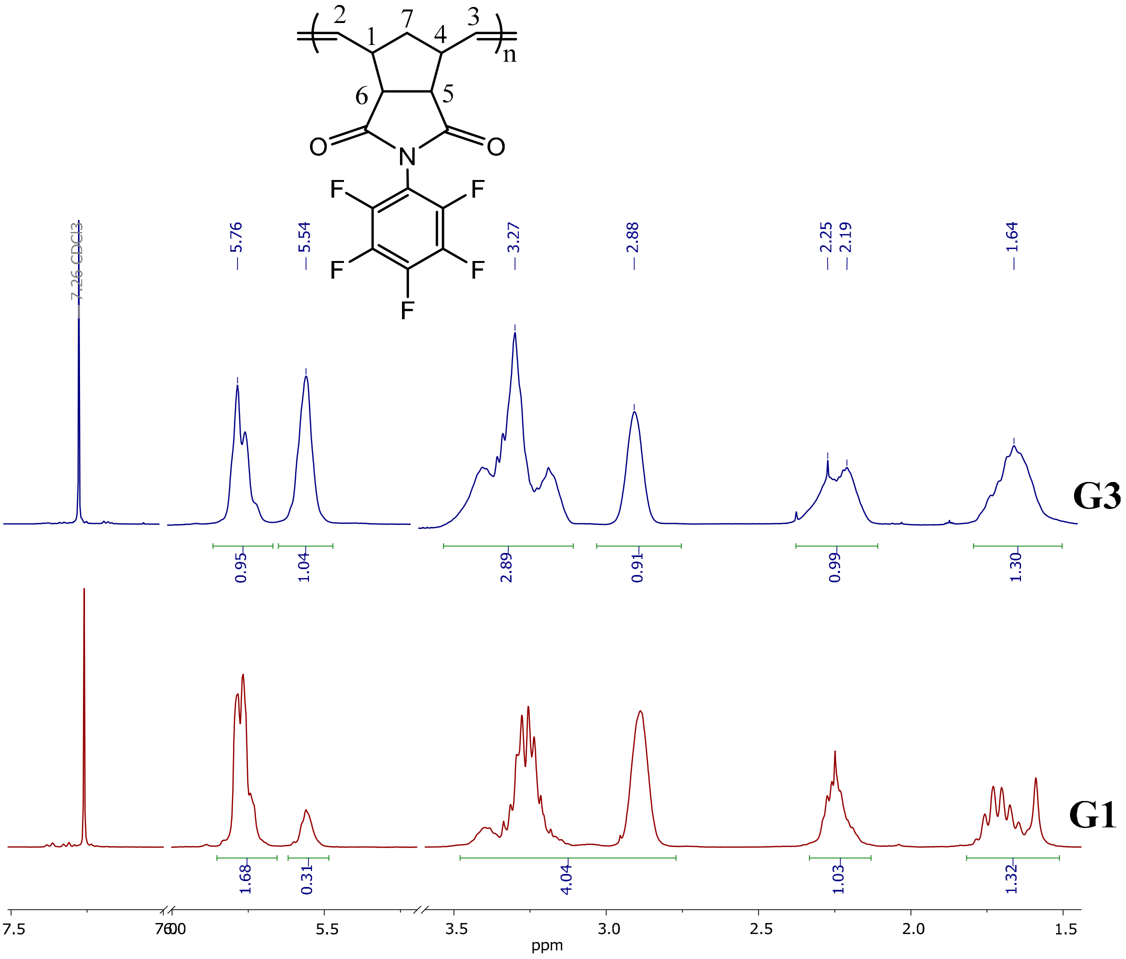

Figure S3. ^{1}H NMR spectra of poly(pentafluorophenyl-norbornene-5,6-dicarboximide) (PNBFD) obtained on the G1 and G3 catalysts at room temperature.

^{1}H NMR (400.1 MHz, $CDCl_3$) δ, ppm: 5.76 (CH=CH, *trans*), 5.54 (CH=CH, *cis*), 3.27 (3H), 2.88 (1H), 2.25, 2.19 (1H), 1.64 (1H).

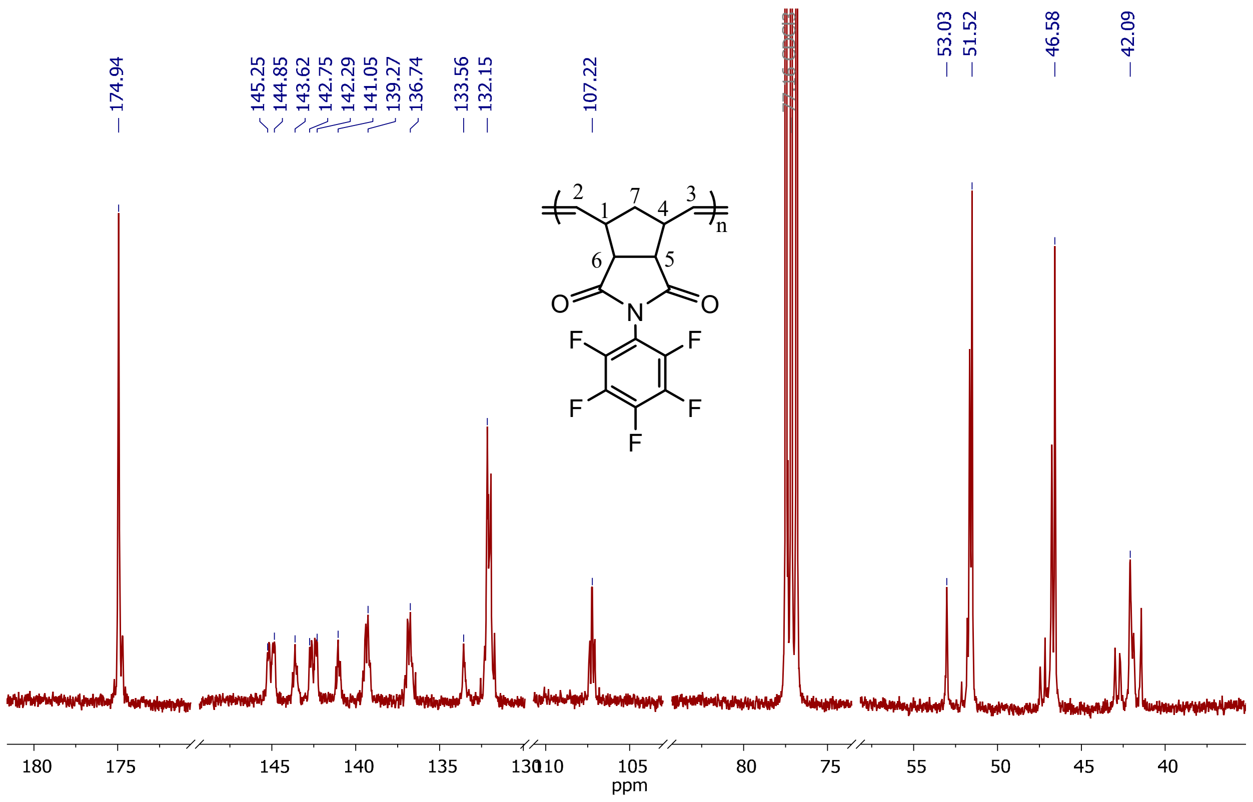

Figure S4. ^{13}C NMR spectrum of poly(pentafluorophenyl-norbornene-5,6-dicarboximide) (PNBFD) at room temperature.

^{13}C NMR (100.6 MHz, $CDCl_3$) δ, ppm: 174.94 (C=O), 145.25, 144.85, 143.62, 142.75, 142.29, 141.05, 139.27, 136.74 (CF), 133.56 (C=C, *cis*), 132.15 (C=C, *trans*), 107.22 (C-N), 53.03 ($C^{1,4}$, *cis*) 51.52 ($C^{1,4}$, *trans*), 46.58, 42.09.

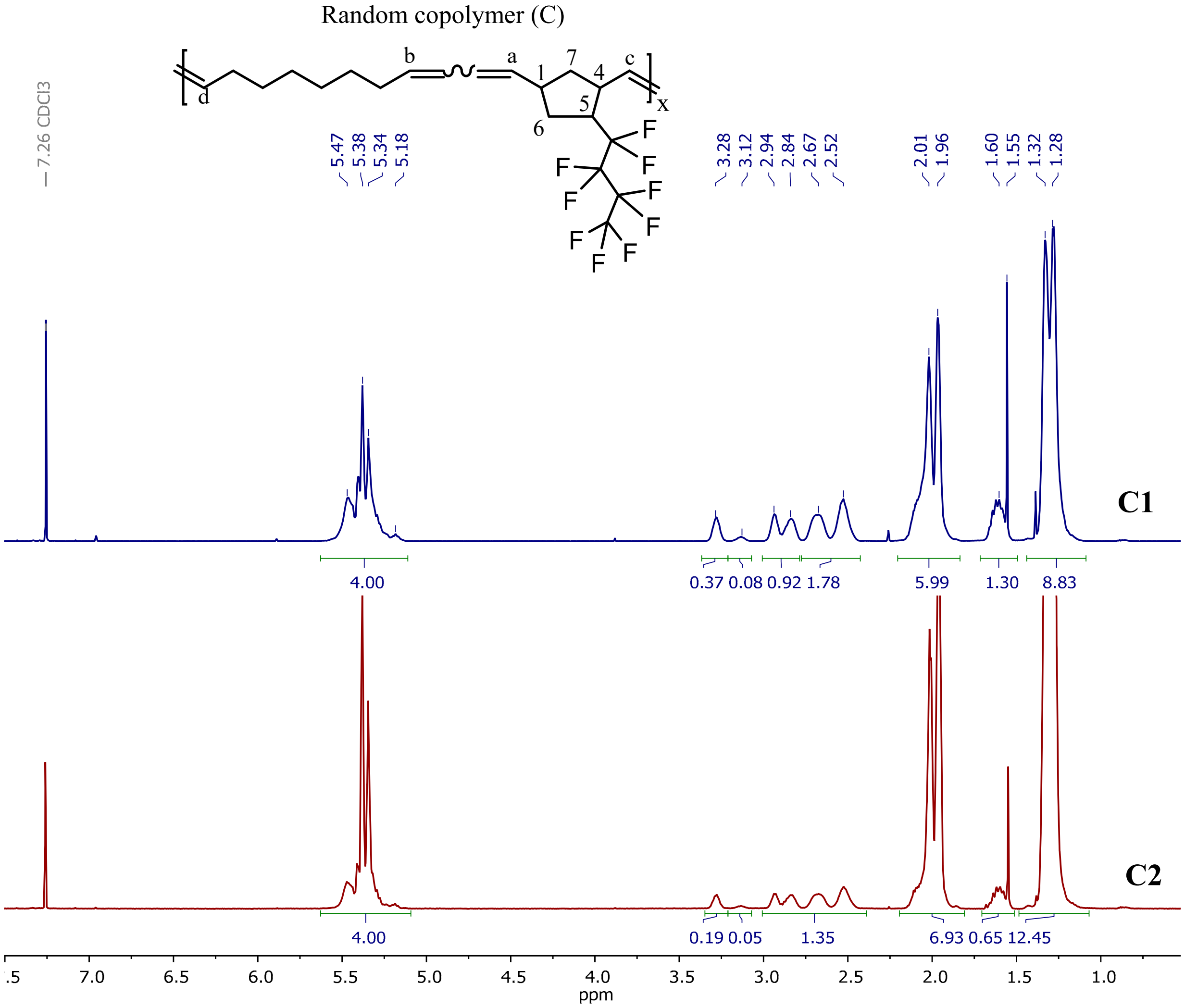

Figure S5. ^{1}H NMR spectra of NBF–COE random C1 and C2 copolymers at room temperature.

^{1}H NMR (400.1 MHz, $CDCl_3$) δ, ppm: 5.47, 5.38, 5.34, 5.18 (HC=CH, PNBF, PCOE), 3.28 (HC^5, *cis*, *endo,* PNBF), 3.12 (HC^5, *cis*, *exo,* PNBF), 2.94 (HC^5, *trans*, *endo,* PNBF), 2.84 (HC^5, *trans*, *exo,* PNBF), 2.67 ($HC^{1,4}$, *cis*, PNBF), 2.52 ($HC^{1,4}$, *trans,* PNBF), 2.01, 1.96 ((H_2C^6, PNBF; 4H, PCOE), 1.60, 1.55 (H_2C^7, PNBF), 1.32, 1.28 (H_2C^7, PNBF; 8H, PCOE).

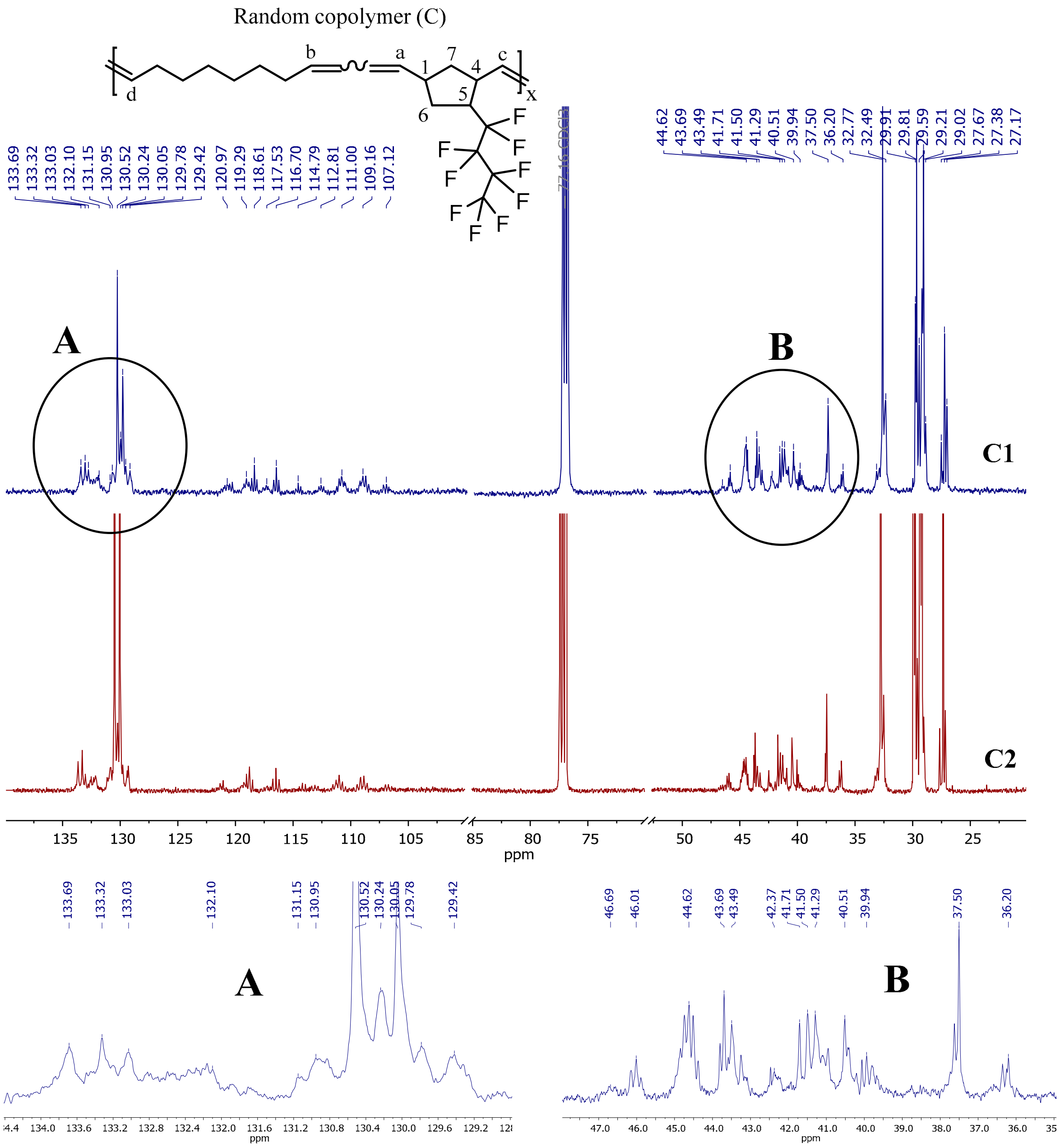

Figure S6. ^{13}C NMR spectra of NBF–COE random C1 and C2 copolymers at room temperature.

^{13}C NMR (100.6 MHz, $CDCl_3$) δ, ppm: 133.69, 133.32, 133.03, 132.10, 131.15, 130.95 (C=C, PNBF), 130.52 (C=C *trans*, PCOE), 130.24 (C=C, PNBF), 130.05 (C=C *cis*, PCOE), 129.78, 129.42 (C=C, PNBF), 120.97, 119.29, 118.61, 117.53, 116.70, 114.79, 112.81, 111.00, 109.16, 107.12 (C-F), 46.69 (C^5, *exo*, *cis*, PNBF), 46.01 (C^5, *exo*, *trans*, PNBF), 44.62 (C^5, *endo*, *cis/trans*, PNBF), 43.69, 43.49 (C^1, PNBF), 42.37, 41.47, 41.50, 41.29, 40.51, 39.94 ($C^{4,7}$, PNBF), 37.50, 36.20 ($C^{1,4}$, PNBF), 32.77, 32.49 (C^6, PNBF; CH_2, PCOE), 29.91, 29.81, 29.59, 29.21, 29.02, 27.67, 27.38, 27.17 (CH_2, PCOE).

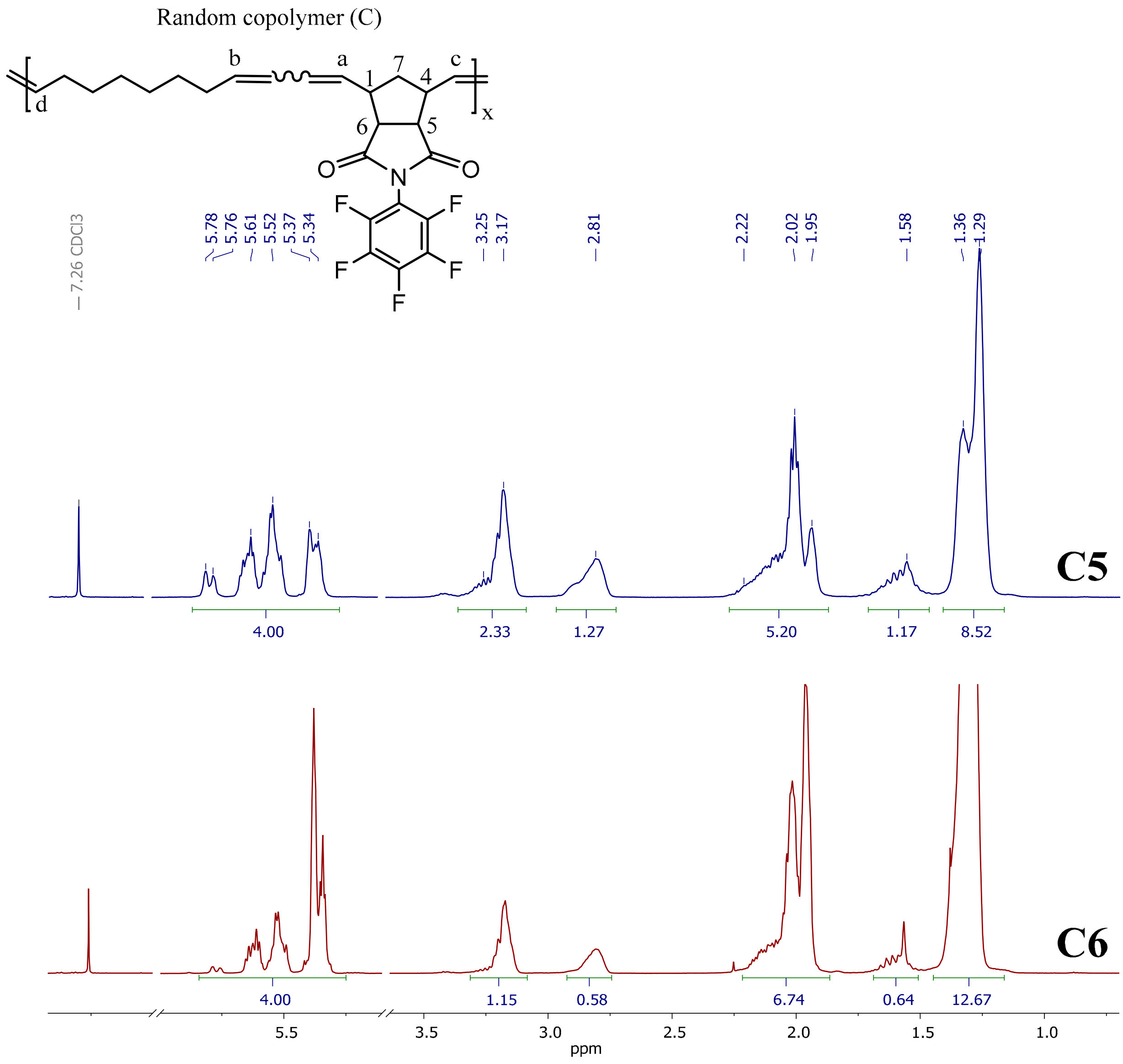

Figure S7. ^{1}H NMR spectra of NBFD–COE random C5 and C6 copolymers at room temperature.

^{1}H NMR (400.1 MHz, $CDCl_3$) δ, ppm: 5.78 ($HC^{a}=C^{c}H$ *trans*, PNBFD), 5.61 (heterodyad $\mathbf{HC^{a}}=C^{b}H$), 5.52 ($HC^{a}=C^{c}H$ *cis*, PNBFD; heterodyad $HC^{a}=\mathbf{C^{b}H}$), 5.37 ($HC^{b}=C^{d}H$ *trans*, PCOE), 5.34 ($HC^{b}=C^{d}H$ *cis*, PCOE), 3.25-3.17 (PNBFD), 2.81 (PNBFD), 2.22, 2.02 (PNBFD), 1.95 (PCOE), 1.58 (PNBFD), 1.36, 1.29 (PCOE, PNBFD).

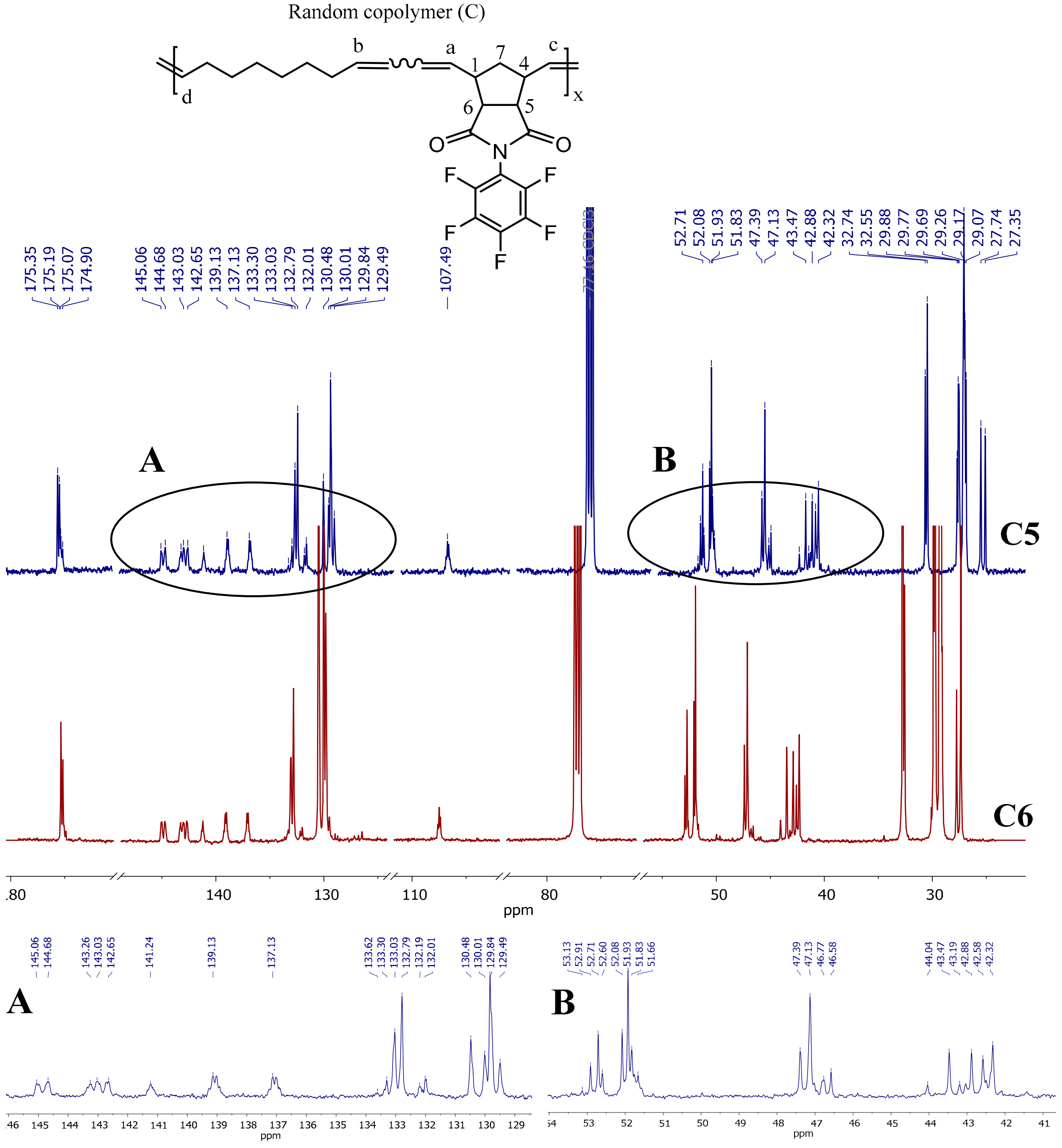

Figure S8. ^{13}C NMR spectra of NBFD–COE random C5 and C6 copolymers at room temperature.

^{13}C NMR (100.6 MHz, $CDCl_3$) δ, ppm: 175.35, 175.19, 175.07, 174.90 (C=O), 145.06, 144.68, 143.26, 143.03, 142.65, 141.24, 139.13, 137.13 (C-F), 133.62 (HC^a=C^cH *cis*, PNBFD), 133.30, 133.03, 132.79 (heterodyad $\mathbf{C^a}$=C^b), 132.19, 132.01 (HC^a=C^cH *trans*, PNBFD), 130.48 (HC^b=C^dH *trans*, PCOE), 130.01 (HC^b=C^dH *cis*, PCOE), 129.84, 129.49 (heterodyad C^a=$\mathbf{C^b}$), 107.49 (C-N), 53.13, 52.91, 52.71, 52.60 ($C^{1,4}$, *cis,* PNBFD*)*, 52.08, 51.93, 51.83, 51.66 ($C^{1,4}$, *trans*, PNBFD), 47.39, 47.13, 46.77, 46.58, 44.04, 43.47, 43.19, 42.88, 42.58, 42.32 ($C^{5,6,7}$, PNBFD), 32.74 , 32.55, 29.88, 29.77, 29.69, 29.26, 29.17, 29.07, 27.74, 27.35 (CH_2, PCOE).

The average lengths of NBFD and COE blocks in the copolymers, L_{NBFD} and L_{COE}, were calculated from the integral intensities of homo- (NBFD–NBFD (133.62, 132.19, 132.01 ppm), COE–COE

(130.48, 130.01 ppm)) and hetero- (NBFD–COE (133.30, 133.03, 132.79 ppm), COE–NBFD (129.84, 129.49)) dyad signals in ^{13}C NMR spectra:

$$L_{NBFD} = \frac{I\,(C = C_{NBFD-COE}) + I(C = C_{NBFD-NBFD})}{I(C = C_{NBFD-COE})}$$

$$L_{COE} = \frac{I\,(C = C_{COE-NBFD}) + I(C = C_{COE-COE})}{I(C = C_{COE-NBFD})}$$

Multiblock copolymer (M)

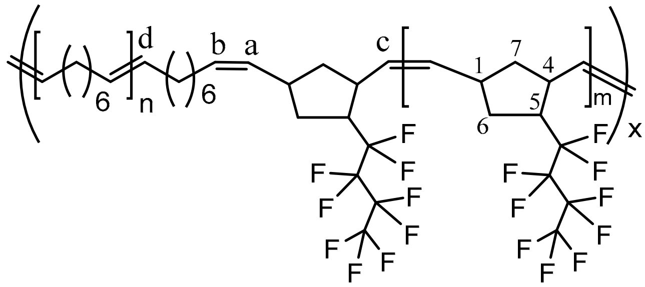

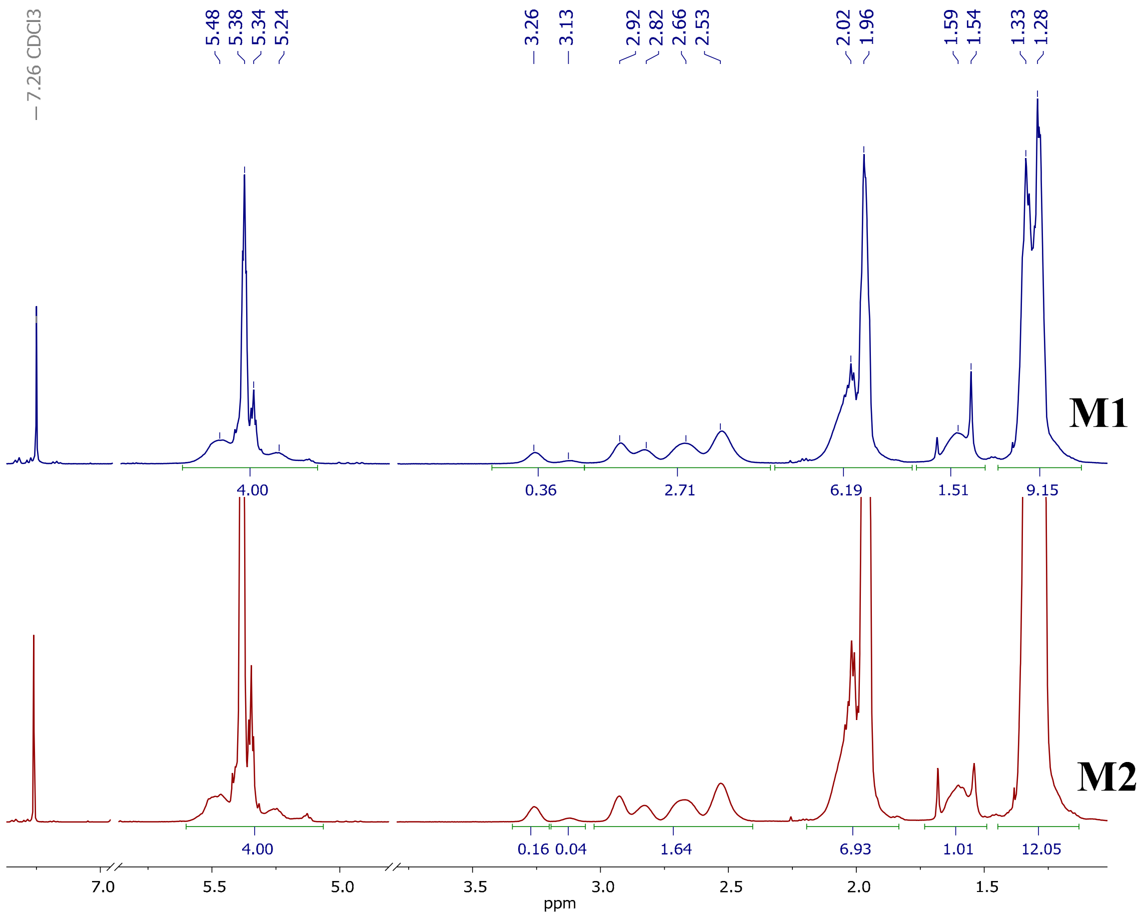

Figure S9. ^{1}H NMR spectra of NBF–COE multiblock M1 and M2 copolymers at room temperature.

^{1}H NMR (400.1 MHz, CDCl3) δ, ppm: 5.48, 5.38, 5.34, 5.24 (HC=CH, PNBF, PCOE), 3.26 (HC^5, *cis*, *endo*, PNBF), 3.13 (HC^5, *cis*, *exo*, PNBF), 2.92 (HC^5, *trans*, *endo*, PNBF), 2.82 (HC^5, *trans*, *exo*, PNBF), 2.66 (HC^{1,4}, *cis*, PNBF), 2.53 (HC^{1,4}, *trans*, PNBF), 2.02, 1.96 ((H_2C^6, PNBF; 4H, PCOE), 1.59, 1.54 (H_2C^7, PNBF), 1.33, 1.28 (H_2C^7, PNBF; 8H, PCOE).

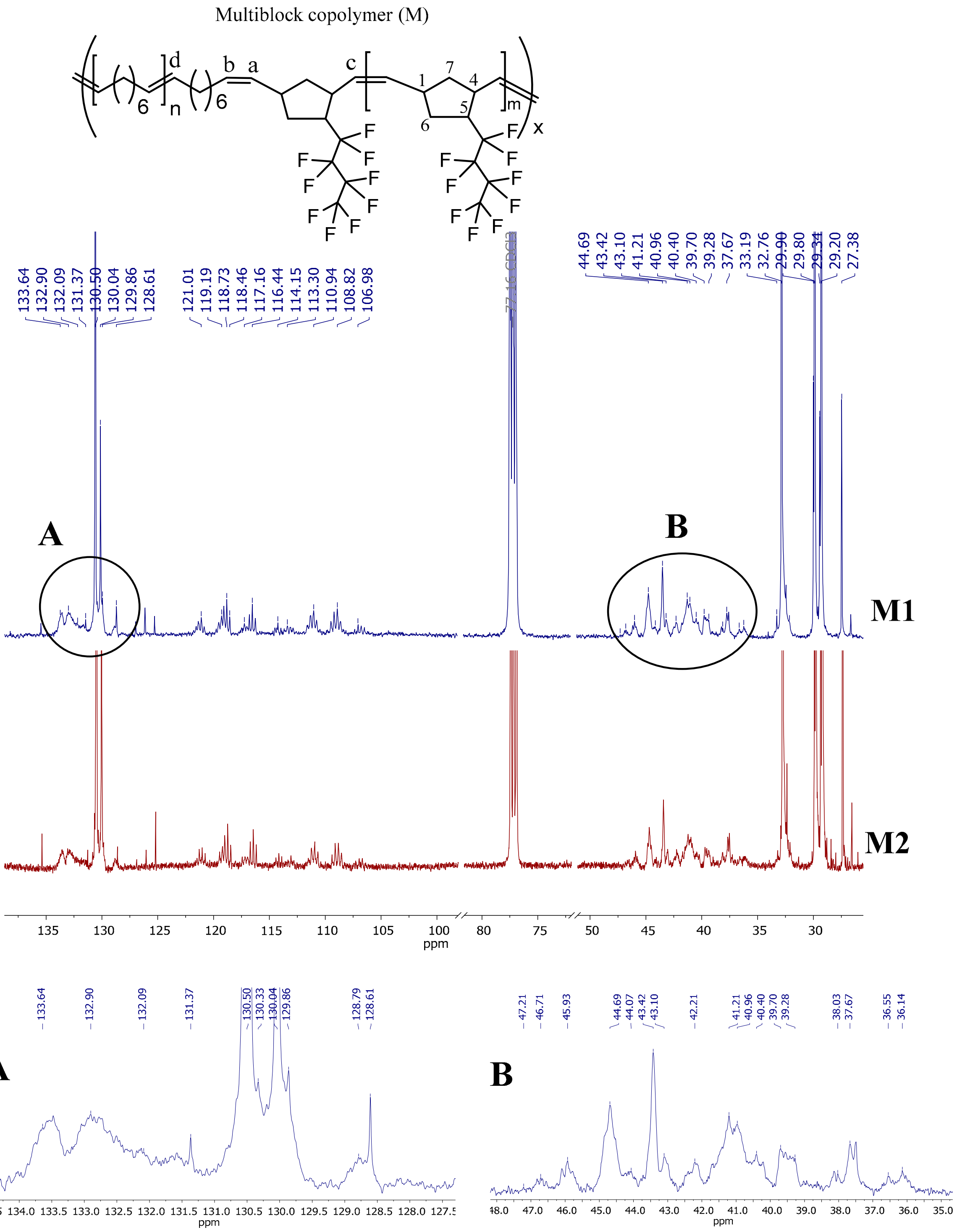

Figure S10. ^{13}C NMR spectra of NBF–COE multiblock M1 and M2 copolymers at room temperature.

^{13}C NMR (100.6 MHz, $CDCl_3$) δ, ppm: 133.64, 132.90, 132.09, 131.37 (C=C, PNBF), 130.50 (C=C *trans*, PCOE), 130.33 (C=C, PNBF), 130.04 (C=C *cis*, PCOE), 129.86, 128.79, 128.61 (C=C, PNBF), 121.01, 119.19, 118.73, 118.46, 117.16, 116.44, 114.15, 113.30, 110.94, 108.82, 106.98 (C-F), 47.21 (C^5, *exo, cis,* PNBF), 46.71 (C^5, *exo*, *trans,* PNBF), 45.93 (C^5, *endo, cis/trans,* PNBF), 44.69, 44.07 (C^1, PNBF), 43.42, 43.10 (C^7, PNBF), 42.21, 41.21, 40.96, 40.40, 39.70, 39.28 ($C^{4,7}$, PNBF), 38.03, 37.67, 36.55, 36.14 ($C^{1,4}$, PNBF), 33.19, 32.76, 32.05 (C^6, PNBF; CH_2, PCOE), 29.90, 29.80, 29.34, 29.20, 27.38 (CH_2, PCOE).

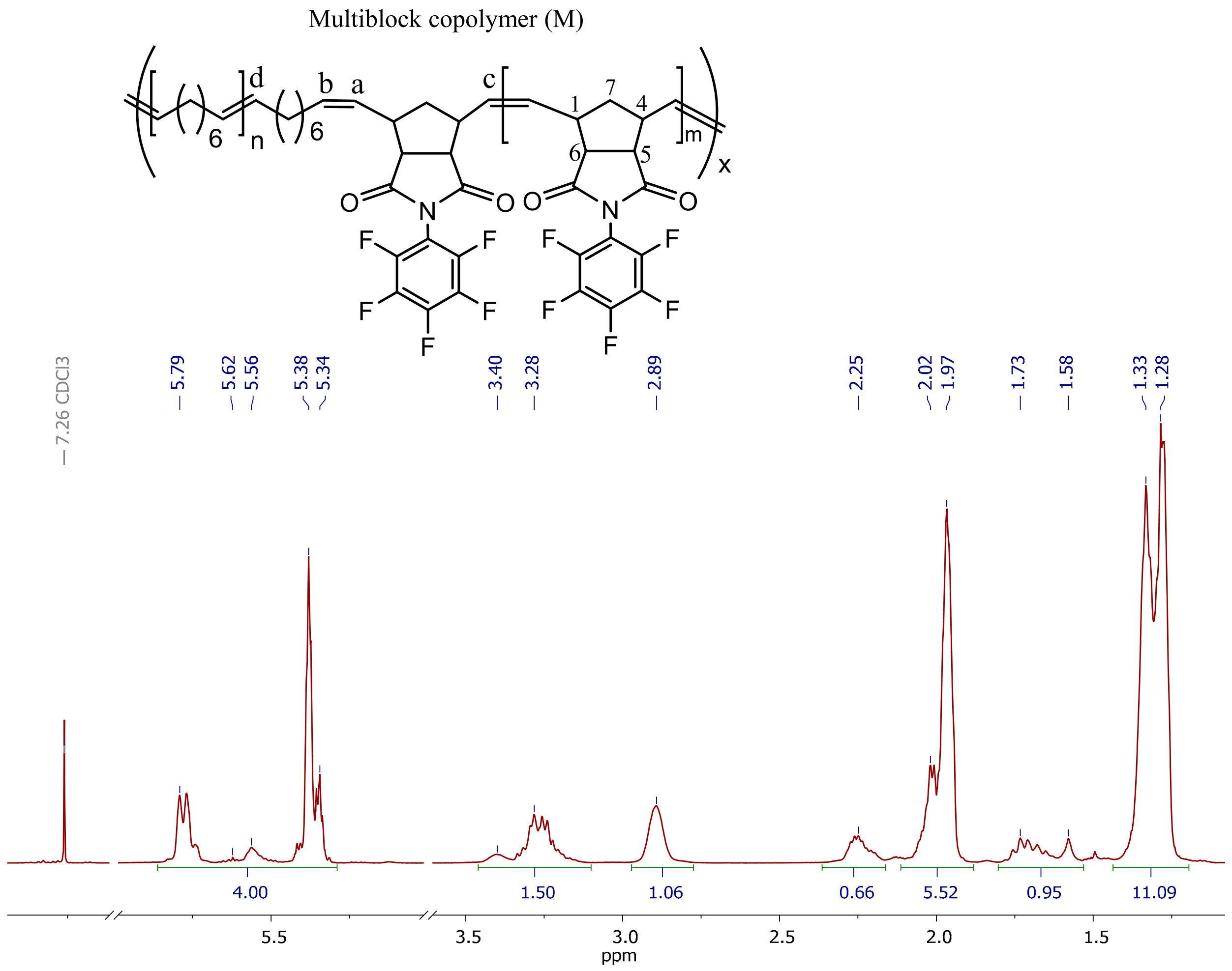

Figure S11. ^{1}H NMR spectrum of NBFD–COE multiblock M4 copolymer at room temperature.

^{1}H NMR (400.1 MHz, CDCl3) δ, ppm: 5.79 (HC^{a}=C^{c}H *trans*, PNBFD), 5.62 (heterodyad **HC^{a}**=C^{b}H), 5.56 (HC^{a}=C^{c}H *cis*, PNBFD; heterodyad HC^{a}=**C^{b}H**), 5.38 (HC^{b}=C^{d}H *trans*, PCOE), 5.34 (HC^{b}=C^{d}H *cis*, PCOE), 3.40, 3.28, 2.89 (PNBFD), 2.25, 2.02 (PNBFD), 1.97 (PCOE), 1.73, 1.58 (PNBFD), 1.33, 1.28 (PCOE, PNBFD).

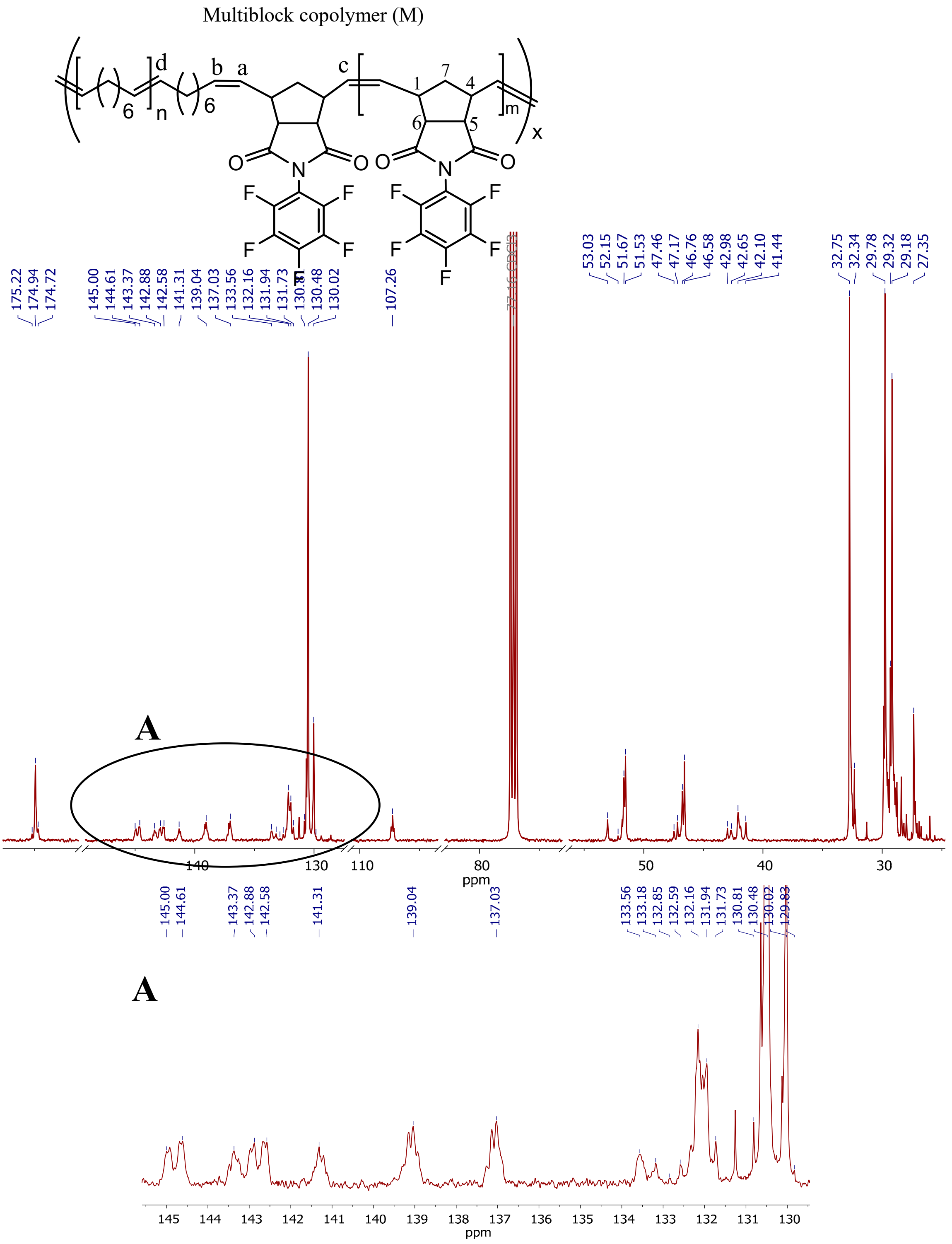

Figure S12. ^{13}C NMR spectrum of NBFD–COE multiblock M4 copolymer at room temperature.

^{13}C NMR (100.6 MHz, $CDCl_3$) δ, ppm: 175.22, 174.94, 174.72 (C=O), 145.00, 144.61, 143.37, 142.88, 142.58, 141.31, 139.04, 137.03 (C-F), 133.56, 133.18 (HC^a=C^cH *cis*, PNBFD), 132.85 (heterodyad $\mathbf{C^a}$=C^b), 132.59, 132.16, 131.94, 131.73 (HC^a=C^cH *trans*, PNBFD), 130.48 (HC^b=C^dH *trans*, PCOE), 130.02 (HC^b=C^dH *cis*, PCOE), 129.83 (heterodyad C^a=$\mathbf{C^b}$), 107.26 (C-N), 53.03 ($C^{1,4}$, *cis,* PNBFD), 52.15, 51.67, 51.53 ($C^{1,4}$, *trans*, PNBFD), 47.46, 47.17, 46.76, 46.58, 42.98, 42.65, 42.10, 41.44 ($C^{5,6,7}$, PNBFD), 32.75, 32.34, 29.78, 29.32, 29.18, 28.77, 27.35 (CH_2, PCOE).

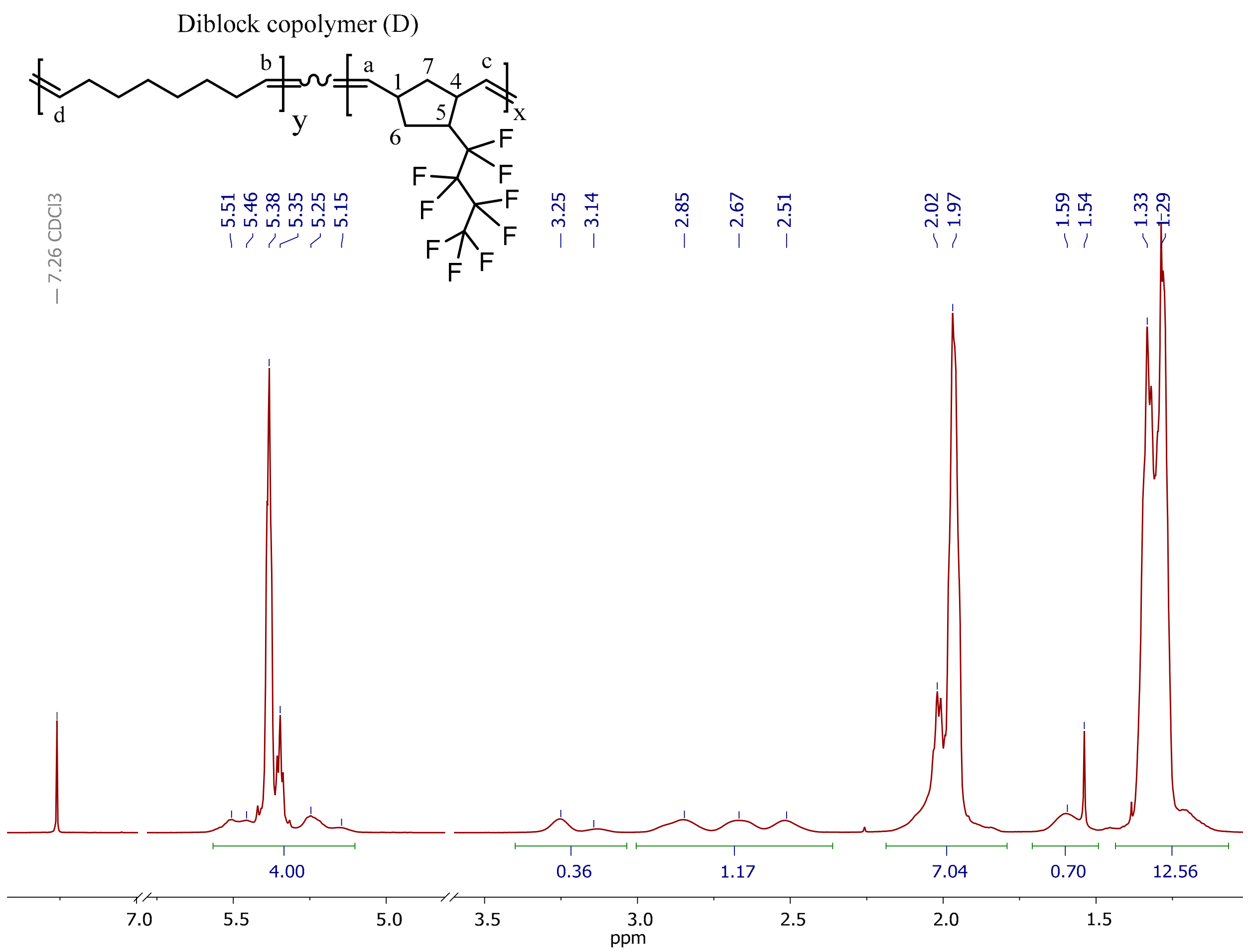

Figure S13. ^{1}H NMR spectrum of NBF–COE diblock D1 copolymer at room temperature.

^{1}H NMR (400.1 MHz, CDCl3) δ, ppm: 5.51, 5.46, 5.38, 5.35, 5.25, 5.15 (HC=CH, PNBF, PCOE), 3.25 (HC^5, *cis*, *endo,* PNBF), 3.14 (HC^5, *cis*, *exo,* PNBF), 2.85 (HC^5, *trans*, *endo/exo,* PNBF*)*, 2.67 (HC^{1,4}, *cis*, PNBF), 2.51 (HC^{1,4}, *trans,* PNBF), 2.02, 1.97 ((H_2C^6, PNBF; 4H, PCOE), 1.59, 1.54 (H_2C^7, PNBF), 1.33, 1.29 (H_2C^7, PNBF; 8H, PCOE).

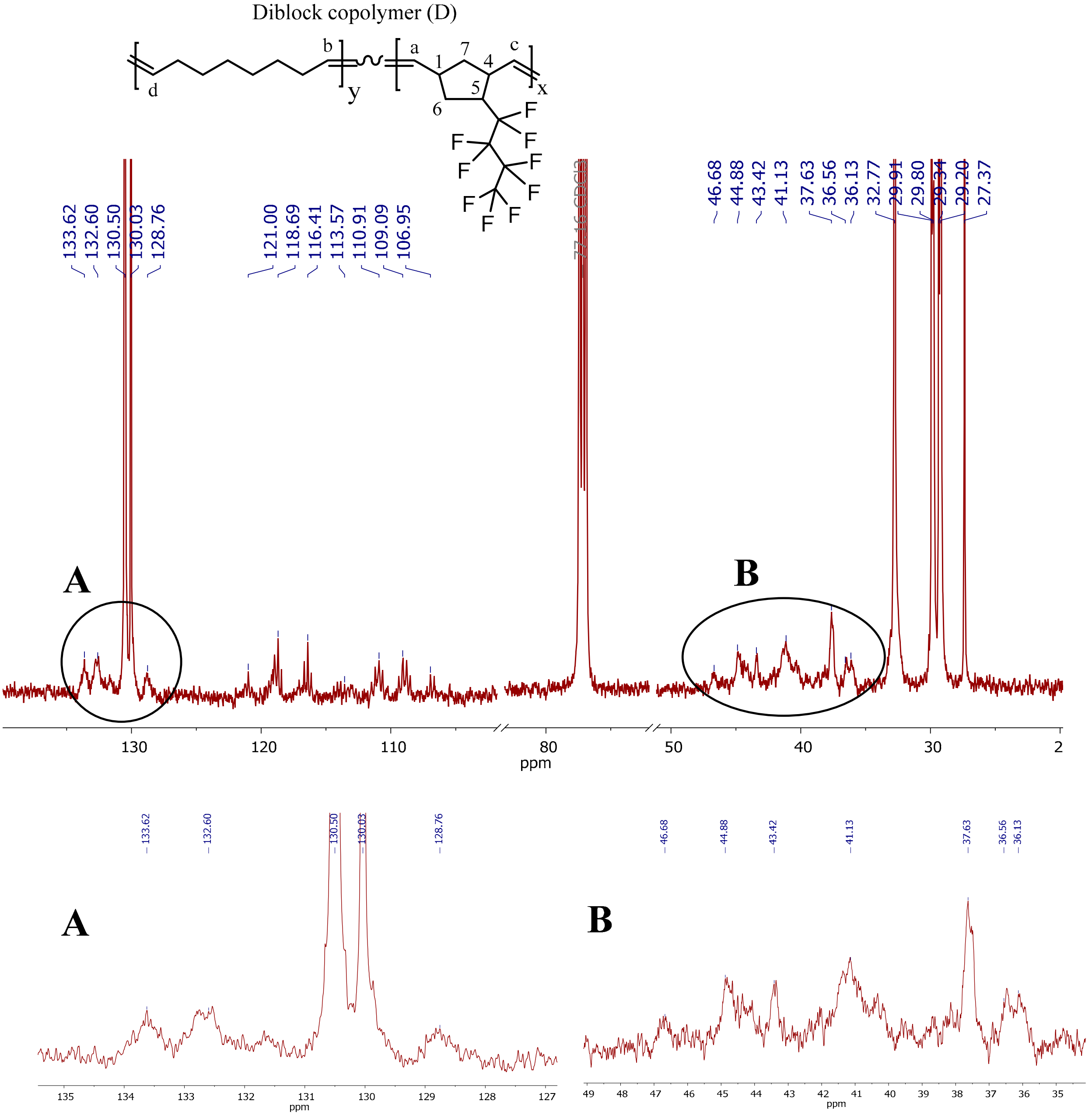

Figure S14. ^{13}C NMR spectrum of NBF–COE diblock D1 copolymer at room temperature.

^{13}C NMR (100.6 MHz, $CDCl_3$) δ, ppm: 133.62, 132.60 (C=C, PNBF), 130.50 (C=C *trans*, PCOE), 130.03 (C=C *cis*, PCOE), 128.76 (C=C, PNBF), 121.00, 118.69, 116.41, 113.57, 110.91, 109.09, 106.95 (C-F), 46.68 (C^5, *exo*, *trans,* PNBF), 44.88, 43.42 ($C^{1,5,7}$, PNBF), 41.13 ($C^{4,7}$, PNBF), 37.63, 36.56, 36.13 ($C^{1,4}$, PNBF), 32.77 (C^6, PNBF; CH_2, PCOE), 29.91, 29.80, 29.34, 29.20, 27.37 (CH_2, PCOE).

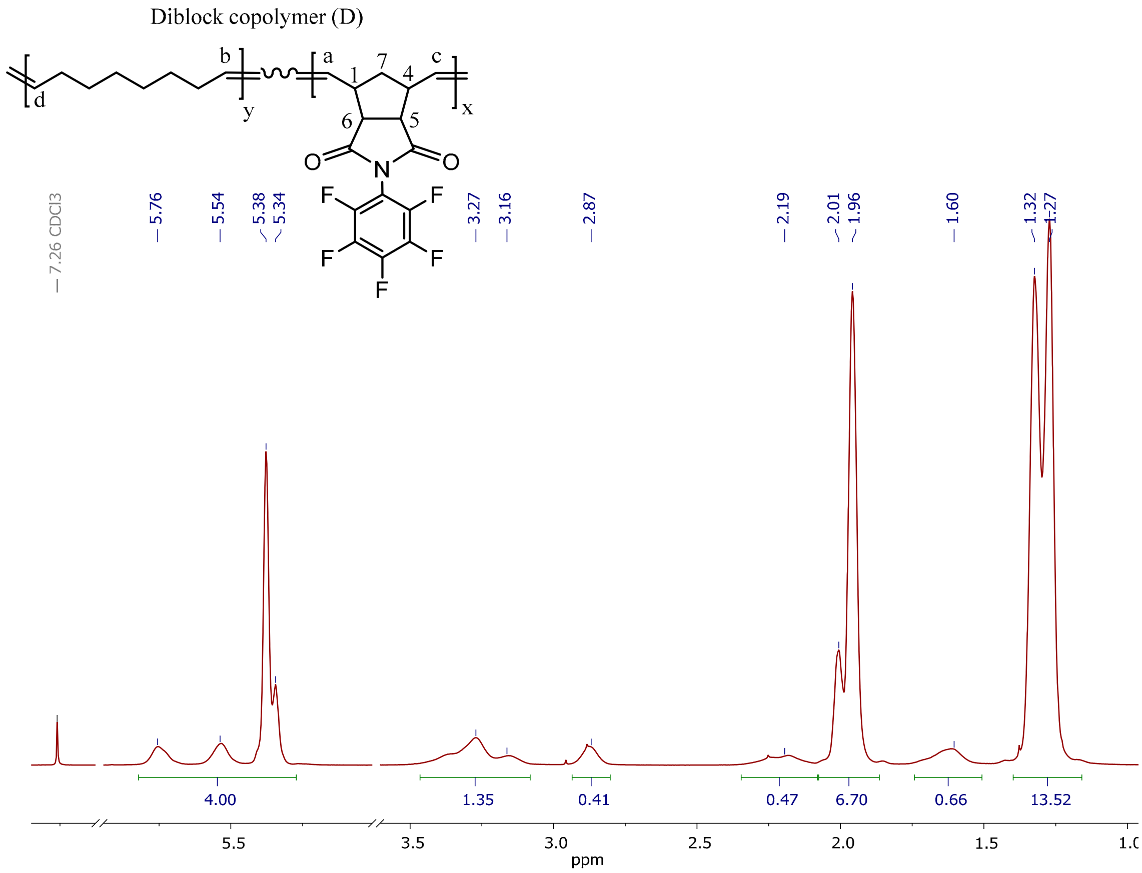

Figure S15. ^{1}H NMR spectrum of NBFD–COE diblock D2 copolymer at room temperature.

^{1}H NMR (400.1 MHz, CDCl3) δ, ppm: 5.76 ($HC^a{=}C^cH$ *trans*, PNBFD), 5.54 ($HC^a{=}C^cH$ *cis*, PNBFD), 5.38 ($HC^b{=}C^dH$ *trans*, PCOE), 5.34 ($HC^b{=}C^dH$ *cis*, PCOE), 3.27, 3.16, 2.87 (PNBFD), 2.19, 2.01 (PNBFD), 1.96 (PCOE), 1.60 (PNBFD), 1.32, 1.27 (PCOE, PNBFD).

Diblock copolymer (D)

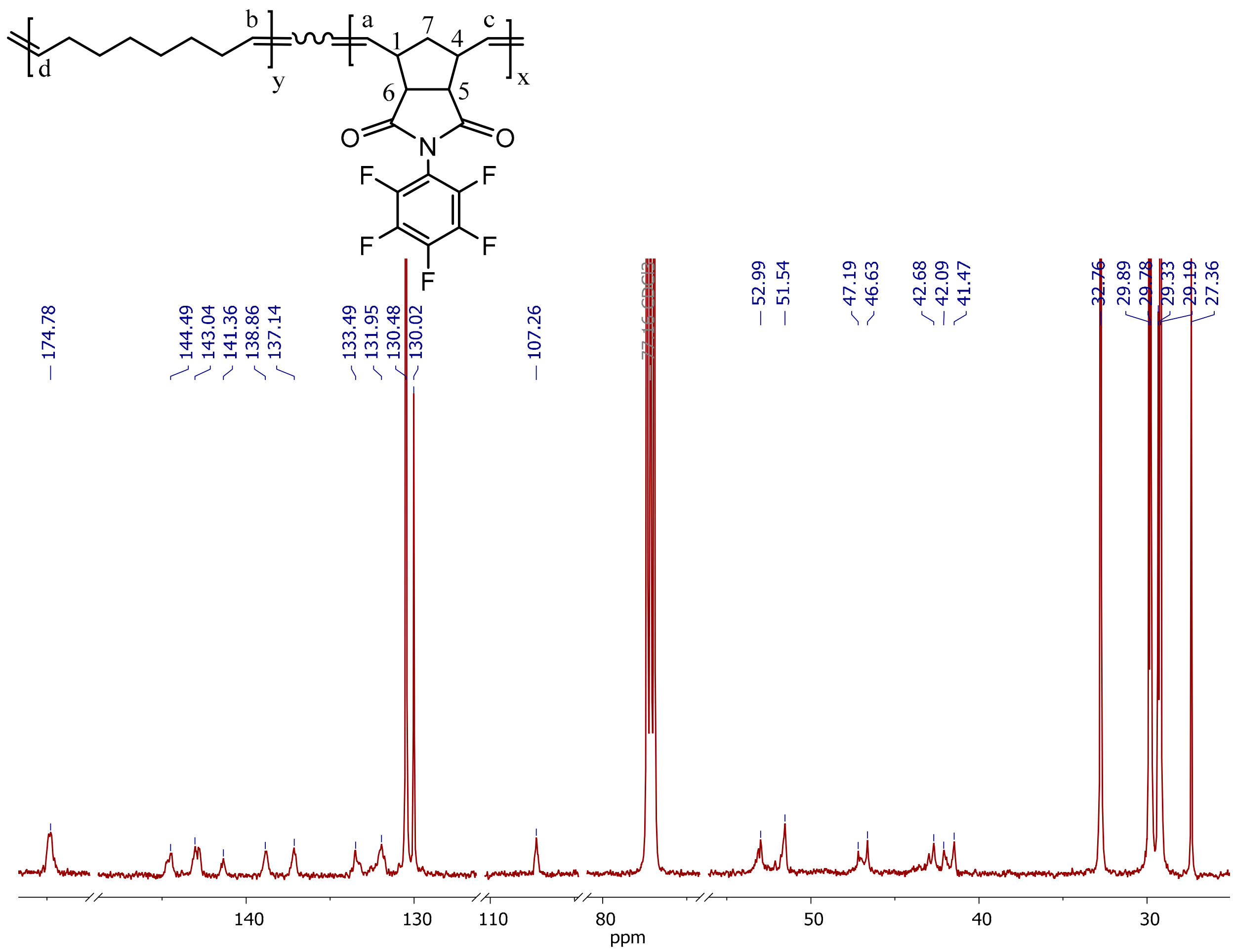

Figure S16. ^{13}C NMR spectrum of NBFD–COE diblock D2 copolymer at room temperature.

^{13}C NMR (100.6 MHz, $CDCl_3$) δ, ppm: 174.78 (C=O), 144.49, 143.04, 141.36, 138.86, 137.14 (C-F), 133.49 ($HC^a{=}C^cH$ *cis*, PNBFD), 131.95 ($HC^a{=}C^cH$ *trans*, PNBFD), 130.48 ($HC^b{=}C^dH$ *trans*, PCOE), 130.02 ($HC^b{=}C^dH$ *cis*, PCOE), 107.26 (C-N), 52.99 ($C^{1,4}$, *cis,* PNBFD), 51.54 ($C^{1,4}$, *trans*, PNBFD), 47.19, 46.63, 42.68, 42.09, 41.47 ($C^{5,6,7}$, PNBFD), 32.76, 29.89, 29.78, 29.33, 29.19, 27.36 (CH_2, PCOE).

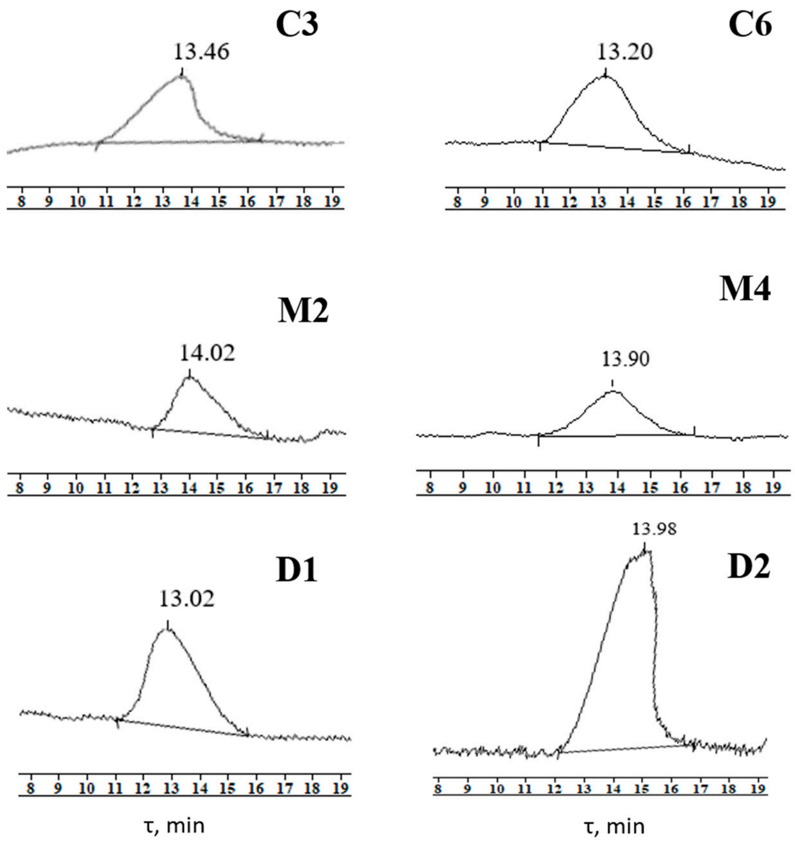

Figure S17. GPC curves of different copolymers NBF and NBFD with COE (copolymer D2 was analyzed from a solution in chloroform).
